# Partial Predictability in Avoidance Acquisition and Expression of Wistar-Kyoto and Sprague-Dawley Rats: Implications for Anxiety Vulnerability in Uncertain Situations

**DOI:** 10.3389/fpsyt.2020.00848

**Published:** 2020-08-19

**Authors:** Daniel Paul Miller, Michael Todd Allen, Richard J. Servatius

**Affiliations:** ^1^ Neuroscience Department, Carthage College, Kenosha, WI, United States; ^2^ Department of Psychiatry, Stress and Motivated Behavior Institute, Upstate Medical University, Syracuse, NY, United States; ^3^ School of Psychological Sciences, University of Northern Colorado, Greeley, CO, United States; ^4^ Department of Veterans Affairs, Syracuse Veterans Affairs Medical Center, Syracuse, NY, United States

**Keywords:** avoidance, behavioral inhibition, expectancy, partial reinforcement, Wistar-Kyoto (WKY) rat

## Abstract

Individual differences or vulnerabilities must exist which bias some individuals toward psychopathology while others remain resilient in the face of trauma. Recent work has studied the effects of uncertainty on individuals expressing behavioral inhibition (BI). The current study extended this work with uncertainty to Wistar Kyoto (WKY) rats which are a behaviorally inhibited inbred strain that models learning vulnerabilities for anxiety disorders and posttraumatic stress disorder (PTSD). WKY rats exhibit superior avoidance performance in a signaled bar press avoidance task in which a tone conditioned stimulus (CS) signals a foot shock unconditional stimulus (US) when compared with non-inhibited Sprague-Dawley (SD) rats. In addition, WKY rats express enhanced eyeblink conditioning. Recent work with behaviorally inhibited humans has indicated that this enhanced eyeblink conditioning is more evident in conditions that insert CS- or US-alone trials into CS-US paired training, resulting in uncertain and suboptimal learning conditions. The current study examined the effects of partial predictability training, in which the CS signaled the US only one-half of the time, on the acquisition and expression of avoidance. Standard training with a fixed 60-s CS which predicted shock on 100% of trials was compared with training in which the CS predicted shock on 50% of trials (partial predictability) using a pseudorandom schedule. As expected, WKY rats acquired avoidance responses faster and to a greater degree than SD rats. Partial predictability of the US essentially reduced SD rats to escape responding. Partial predictability also reduced avoidance in WKY rats; however, adjusting avoidance rates for the number of potential pairings of the CS and US early in training suggested a similar degree of avoidance expression late in the last session of training. Enhanced active avoidance expression, even in uncertain learning conditions, can be interpreted as behaviorally inhibited WKY rats responding to the expectancy of the shock by avoiding, whereas non-inhibited SD rats were responding to the presence of the shock by escaping. Future work should explore how WKY and SD rats as well as behaviorally inhibited humans acquire and extinguish avoidance responses in uncertain learning situations.

## Introduction

Since most individuals who experience a traumatic event do not go on to develop post-traumatic stress disorder (PTSD), individual differences or vulnerabilities must exist that bias some individuals toward psychopathology while others remain resilient in the face of trauma. One vulnerability for PTSD and anxiety disorders is an enhanced reactivity to uncertainty (1–4). For example, pre-trauma intolerance of uncertainty predicts PTSD symptoms (5), intolerance of uncertainty is a moderator between worry and PTSD hyperarousal symptoms (6), and intolerance of uncertainty is related to all symptoms of PTSD except re-experiencing (7).

Another such vulnerability that has received much attention is avoidance, in which an individual takes effortful steps to alter the form and/or frequency of events that may be tied to experiences evoking anxiety-like responses (8). Acquisition and/or expression of avoidance is excessive in those with psychopathology, whether motivated by external events or experiences (behavioral avoidance) or internal concepts and affective states (experiential avoidance and emotional avoidance). Moreover, avoidance contributes to the chronicity of these disorders as it prevents the individual from learning that certain situations or stimuli are not or are no longer dangerous. Exaggerated avoidance would be especially maladaptive in uncertain situations in which an aversive event has a low chance to occur. In these situations, the individual may express high levels of avoidance. These avoidance behaviors are maintained by the false belief that the avoidance prevented the aversive event, rather than learning that the aversive event never would have occurred. Thus, exaggerated avoidance in uncertain situations may underlie the development of maladaptive tendencies. Avoidance, in its various forms, is a core feature of anxiety disorders, posttraumatic stress disorder (PTSD), and obsessive-compulsive disorder (OCD) and individual differences in the tendency to express avoidance to an excessive degree is a source of vulnerability to develop these psychopathologies.

Behaviorally inhibited temperament is one such avoidant tendency found to be related to anxiety disorders (9–11), OCD (12), and PTSD (13). Behavioral inhibition (BI) involves extreme withdrawal in the face of novel social and nonsocial challenges. Not only can BI be studied in humans, there exists an animal model of BI, the Wistar Kyoto (WKY) rat. WKY rats were originally a normotensive comparison for the spontaneously hypertensive rat (14), but became a strain of interest in its own right owing to stress sensitivity (15, 16). As an animal model of trait BI, WKY rats display reduced open field activity (17–20) and social interactions (21). In addition, WKY rats exhibit enhanced avoidance and associative learning. These findings fit with long held theories that posit that both classical and operant conditioning are mechanisms through which the psychopathologies observed in PTSD are manifested [for review, see (22)]. Specifically, WKY rats acquire active discrete-trial lever press avoidance with a tone conditioned stimulus (CS) and foot shock unconditional stimulus (US) more rapidly and express avoidance to a higher degree than outbred Sprague-Dawley (SD) rats (19, 23, 24) and are more resistant to extinction of avoidance (19, 25). In addition, WKY rats acquired conditioned eyeblink responses faster and to a greater degree than uninhibited SD rats (26–28) and exhibited slower extinction on CS-alone trials as compared to SD controls (26). Behaviorally inhibited individuals also express similar enhanced classical eyeblink conditioning with a tone CS and a corneal air puff US (29–34). This finding was replicated with active duty military (35) and veterans (36, 37) who self-reported PTSD symptoms. Taken together, these animal and human findings support a learning diathesis model for the development of anxiety disorders (38–40).

The effects of uncertainty with behaviorally inhibited individuals have been explored in recent studies, testing this learning diathesis model. Allen and colleagues parametrically manipulated CS and US contingencies in eyeblink conditioning in behaviorally inhibited and noninhibited (NI) college students (32). Those trained with 100% paired trials were compared with two degraded contingencies: 1) CS-alone trials intercalated among paired trials and 2) US-alone trials intercalated among paired trials. For the latter comparison, the distinctiveness of learning differences between BI and NI was enhanced; the intercalated US-alone trials degraded learning in NI individuals, whereas acquisition of the eyeblink conditioned response (CR) was essentially unchanged by unpaired US exposures. This enhancement of eyeblink conditioning with a schedule of partial reinforcement has since been supported by findings that behaviorally inhibited Coast Guard personnel self-reporting PTSD symptoms exhibited more conditioned eyeblinks with a 50% CS-alone schedule of partial reinforcement than personnel without PTSD symptoms (35). Overall, behaviorally inhibited individuals exhibit enhanced classical conditioning in sub-optimal learning situations such as partial reinforcement which involves some degree of uncertainty as to the probability and timing of an aversive stimulus.

When conditions are uncertain or suboptimal, learning biases may also be apparent for the acquisition or expression of avoidance (23, 24). One relatively unexplored topic in the current avoidance literature with translational value to our understanding of anxiety disorders and PTSD is the relative contingency between the CS and US. Although partial reinforcement in avoidance received some study during the latter half of the last century (41–43), the focus of this work was on the effect of partial reinforcement on avoidance extinction rather than acquisition. Given that the standard learning process for avoidance is through the prevention or removal of the US, the presence of the US represents a degrading of the former contingency. In studies of partial reinforcement by Davenport, Olsen, and colleagues (41–44), CS-US paired trials were interspersed with trials in which responses during the CS were ineffective, resulting in delivery of the US. Avoidance responses were acquired with this 50% partial reinforcement schedule, albeit at a somewhat slower rate.

Another way in which the contingency between the CS and the US can be degraded is by having trials where the CS occurs without a subsequent US (i.e., CS-alone trials), thus degrading predictability of the CS with respect to aversive outcomes when avoidance is not expressed. We will distinguish this protocol as *partial predictability* to contrast with *partial reinforcement*. This approach focuses on sensitivity to acquire an expectation of shock, upon which avoidance is then reinforced through US absence. A weakness of this approach is that experience with the degraded contingency is dependent upon the behavior of the subject, that is, the more that avoidance is expressed early in training, the less exposure the subject has to the degraded contingency. However, lever press avoidances are instrumental behaviors acquired through auto-shaping (19, 45), providing a platform to potentially observe learning under partial predictability.

In the current work, we sought to explore the effects of manipulating uncertainty by altering the contingency between the CS and US in avoidance training through partial predictability. Having the CS and US pairings on only one-half of the trials may be more ecologically valid than previous studies using 100% paired training because experiences in the real world are more uncertain. Based on the CS-alone partial reinforcement eyeblink studies with behaviorally inhibited individuals summarized above, it was hypothesized that this schedule would lower avoidance expression of SD rats, but that WKY rats would be less affected by the degraded contingency. That is to say, motivation for WKY rats to express avoidance will remain high in the face of the reduced associative strength between the CS and US. The difference in motivation will also be reflected in continued expression of non-reinforced responses [inter-trial responses (ITRs)] which are akin to anxiety (19). Beyond BI, another possible vulnerability factor for PTSD is female gender. Female WKY rats have been found to express greater enhancements in avoidance learning than males (26, 46–48). Because we were testing the effects of degrading the predictability of the US, we chose to exclusively test females in this initial study to maximize the chances of observing avoidance in this non-optimal learning condition.

## Methods

### Animals

Female SD (n = 29) and WKY (n = 34) rats (30 days of age) were obtained from Envigo (Indianapolis, IN). Rats were housed in pairs until open field testing, after which they were housed in single cages (12:12 light cycles, lights on 0600). All rats had at least 2 weeks to acclimate to their housing environment prior to testing. Rats had access to food and water *ad libitum*. All procedures were approved by the Institutional Animal Care and Use Committee (IACUC) in accordance with AAALAC standards.

### Procedures

#### Open Field Task

Open field testing was evaluated similar to that described previously (49). Briefly, a rat was placed in the center of an open field apparatus on the floor of a dimly lit room. The apparatus had a center circle and the remainder of the field was divided into 12 sectors. Three observers recorded the latency to leave the center circle and the number of sectors entered. These dependent measures were used to ensure counterbalancing of open field activity for the 100% and 50% groups by strain.

#### Escape/Avoidance Training

The materials and apparatus for lever-press escape–avoidance training are described in detail elsewhere (19, 23). Briefly, four otherwise identical operant chambers (Coulbourn Instruments, Holliston, MA) were housed in sound attenuating outer chambers. The operant chambers contained a grid floor individually wired to shockers (Coulbourne Instruments, Holliston, MA). A lever was 10.5 cm above the floor on one side of the chamber. A 14-W house light was located 20.5 cm above the floor grid. A speaker was centered in the ceiling of the sound attenuating chamber 26 cm above the floor of the operant chambers. Graphic State software (Coulbourn Instruments, Holliston, MA) controlled stimulus presentation and timing and was used to compile responses.

Training took place between the hours of 0800 and 1400 every other day of the work week (MWF) for a total of four sessions. Each training session began with a 60-s stimulus-free period. Rats were matched for sector visits in the open field within strain and assigned to the standard or partial predictability protocol. The standard protocol (23) consisted of 20 discrete trials. The CS was a fixed 60-s 1-kHz tone (78 dB). The US was a train of 1.5-mA shocks (0.5 s in duration every 3 s) delivered through the grid floor. Each trial started with the delivery of the CS. A lever press during the CS constituted an avoidance, which prevented the commencement of the shock train and ended the trial upon termination of the CS. If a lever press did not occur during the CS, the shock train (20 shocks maximum) commenced. A lever press during the shock train constituted an escape response and immediately terminated the trial. In the absence of a lever press, the trial ended after 20 shocks had been delivered. The inter-trial interval was 3 min, denoted by a flashing light. The partial predictability protocol was the same as the standard protocol except that on 50% of the trials, shocks were not delivered in the absence of an avoidance lever press. For these trials, the end of the CS terminated the trial and initiated the inter-trial interval. A single pseudorandom order was used for shock and no shock trials, which was the same used by Allen and colleagues (32). The schedule began with a no shock trial with shock and no shock trials randomly delivered with the provision that no more than four occurrences of a single trial type occurred in order.

### Data Collation and Analyses

Statistical Package for the Social Sciences (Version 26, IBM Corporation, Armonk, NY) was used for all statistical analyses. Open field behaviors were characterized by latency to leave the center segment and number of sectors crossed. T-tests for independent groups were used to analyze these data. Each escape/avoidance training session was compiled for responses during the initial 60-s stimulus-free period, number of avoidances, escapes, shocks received, and inter-trial responses (ITRs). Each training session was further divided into two blocks of 10 trials (i.e., the first 10 trials and the last 10 trials) for further within session analysis. In addition, the number of avoidances emitted on the first trial were compiled. Training data were analyzed by repeated measures analyses of variance (ANOVA). For repeated measures, Mauchly’s Test of Sphericity was examined with Greenhouse-Geisser adjustments for degrees of freedom and significance where indicated. All data are expressed as means ± standard error of the mean. Inasmuch as the focus was on avoidance and its expression under standard and partial predictability, rats that did not emit an avoidance response in the four sessions of training were excluded from analysis (one SD-100%, four SD-50%, and one WKY-50%).

## Results

### Open Field

BI tendencies of WKY rats were confirmed by their performance in the open field test. WKY rats took significantly longer to leave the center of the open field apparatus than SD rats, t(46) = 2.6, p = .013. The mean latencies to leave the center of the open field apparatus for the WKY and SD rats were 3.1 ± 0.9 s and 0.6 ± 0.2 s, respectively. WKY rats visited significantly fewer sectors compared to SD rats, t(46) = 8.1, p <.001. The mean number of sectors visited for the WKY and SD rats were 25.9 ± 1.7 and 49.2 ± 2.3, respectively.

### Performance During E/A Training

**Figure 1:**
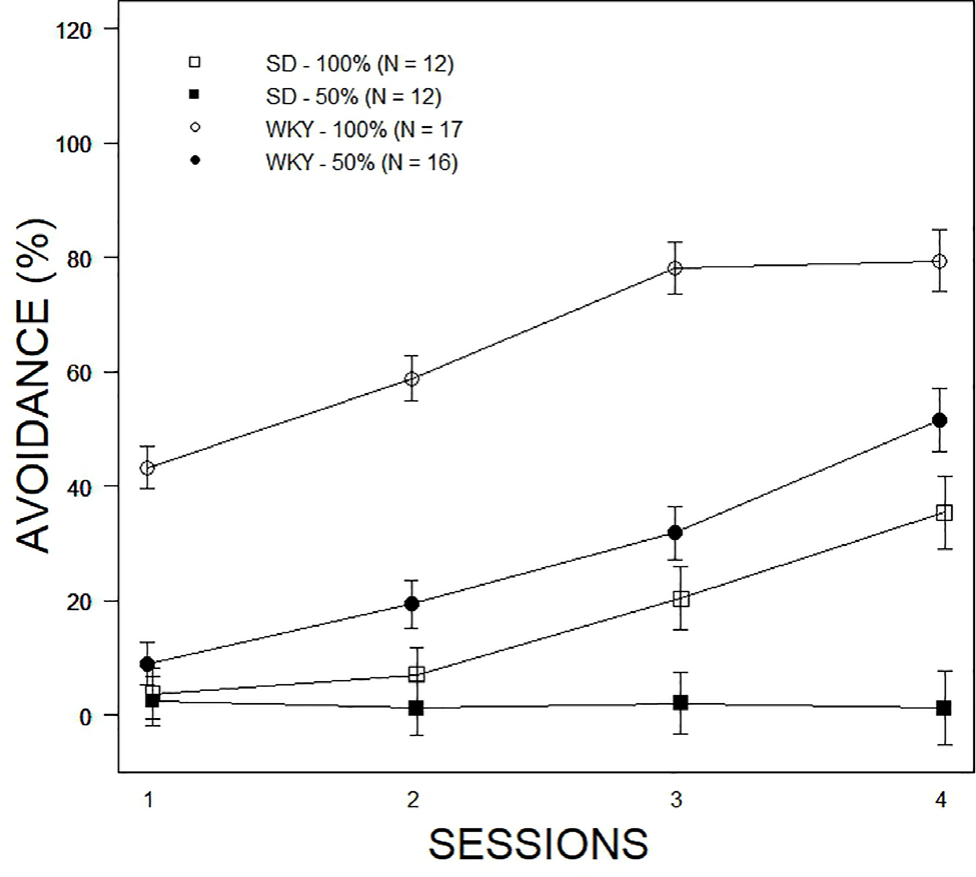
Comparison of avoidance performance as a function of partial predictability in SD and WKY rats. Data are expressed as means ± standard error of the mean. The legend and associated number of rats per group are contained within the figure. All groups showed acquisition of avoidance over the four sessions of training except the SD – 50% group. WKY rats expressed avoidance to a higher degree than SD rats; partial predictability degraded performance.

Overall avoidance acquisition was analyzed with a 2 × 2 × 4 (Strain ×Predictability Schedule × Sessions) mixed-ANOVA. The Strain ×Predictability Schedule × Sessions interaction was significant, F(2.4, 125.7) = 6.5, p = .001 (see **Figure 1**). Avoidance expression increased over sessions in all groups except the SD-50% group, which did not exceed 10%. Expression of avoidance by the WKY-100% group exceeded all other groups in every session. While expression of avoidance increased over sessions in the SD-100% and WKY-50% groups, their rates of expression did not differ appreciably over training. Clearly, the expected Strain difference in avoidance expression was apparent. Moreover, in the face of partial predictability of shock, SD rats almost entirely expressed escape responses. The performance of SD rats in partial predictability precluded further analyses of nonreinforced responses and finer grain analyses of avoidance expression, therefore subsequent analyses only directly compared WKY-100% and WKY-50% groups.

**Figure 2:**
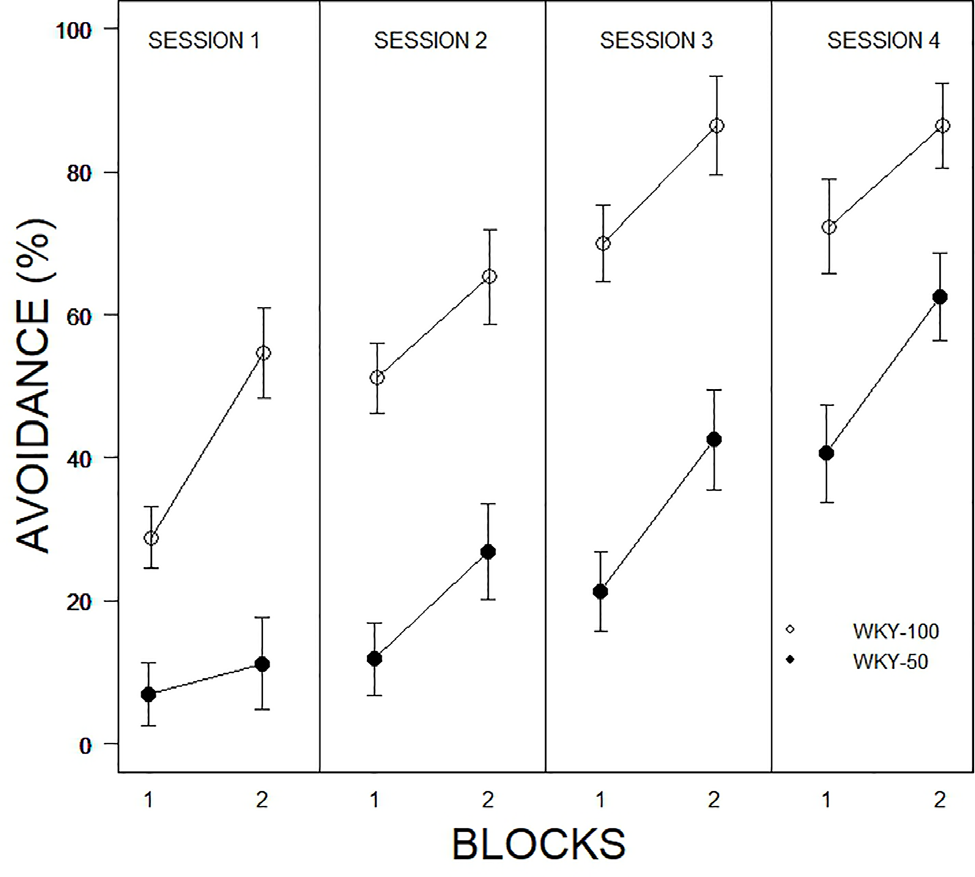
Comparison of avoidance performance as a function of partial predictability in WKY rats as a function of 10-trial blocks over training sessions. Rats trained with full and partial schedules increased avoidance performance over sessions, with avoidance expressed to a higher degree in the second block of each session. WKY rats trained with full predictability (WKY-100) expressed avoidance to a higher degree in every session compared to those trained with partial predictability (WKY-50). Legend is contained within the figure.

**Figure 3:**
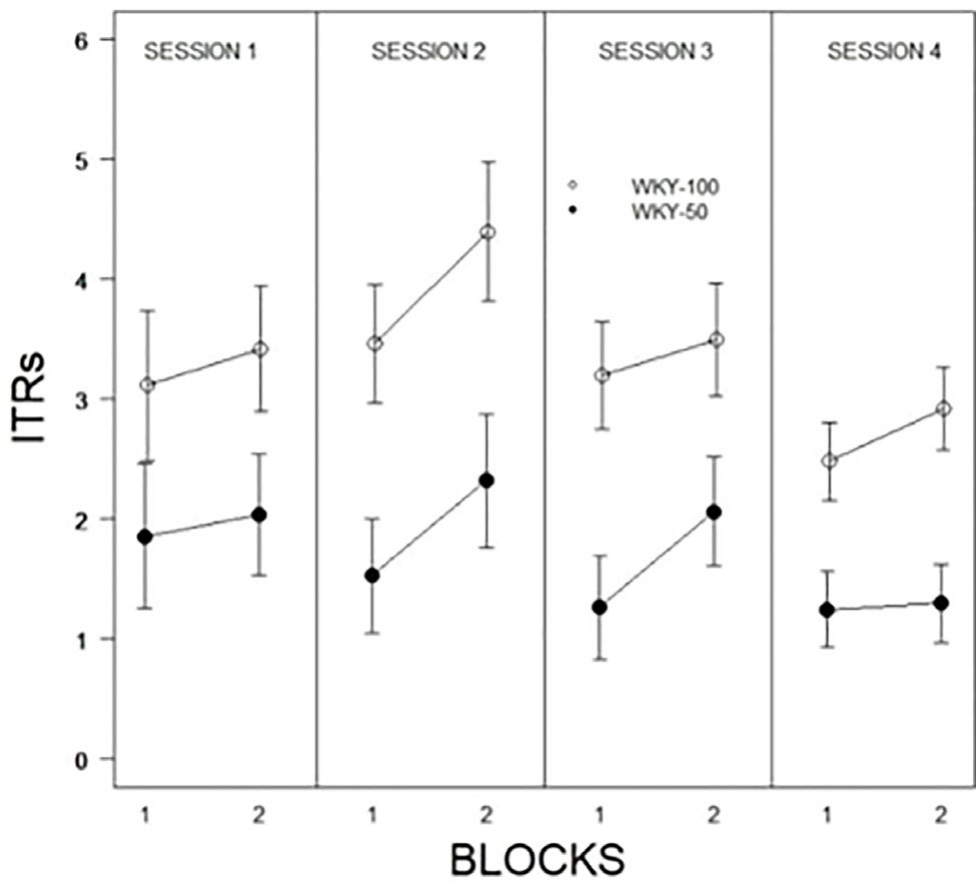
Inter-trial responses (ITRs) of WKY rats trained with full or partial predictability schedules. ITRs were expressed to a greater degree by WKY rats trained with full predictability (WKY-100) compared to partial predictability (WKY-50). ITRs decreased as avoidance was acquired and expressed. Legend is contained within the Figure.

The initial model examined was a 2 × 2 × 10 × 4 (Predictability Schedule × Trial Type × Trial × Session) mixed ANOVA. Trial type was entirely without influence and was dropped from further models. Trial type was spread equally over the first 10 and last 10 trials allowing for two block representations of each session. Therefore, subsequent models were 2 × 2 × 4 (Predictability Schedule × Blocks × Session) mixed ANOVAs. For percent avoidance, there were significant main effects of Blocks, F(1, 31) = 52.7, p < 0.001, Sessions, F(2.4, 75.9) = 46.4, p < 0.001, and Predictability, F(1, 31) = 42.5, all ps < .001 (see **Figure 2**). Expression of avoidance by the WKY-100% group was clearly greater than that of the WKY-50% group. The total number of lever presses during the CS showed a similar pattern, with significant main effects of Blocks, F(1, 31) = 44.5, Sessions, F(2.4, 75.0) = 20.1, and Predictability Schedule, F(1, 31) = 14.2, all ps <.001. A similar model was used to assess the number of responses during the CS; although a single response is sufficient to avoid shock, multiple responses are neither explicitly reinforced nor are they punished. Similar to percent avoidance, main effects of Trials, F(5.8, 140.5) = 7.8, Sessions, F(3, 72) = 16.4, and Predictability Schedule, F(1, 24) = 8.4, were all significant, ps <.004. ITRs are also related to avoidance. The main effects of Session, F(2.4,60.8) = 3.3, Blocks, F(1, 25) = 8.5, and Predictability Schedule, F(1, 25) = 14.1, were all significant, ps <.05. ITRs were emitted: a) with decreasing frequencies over sessions, b) more frequently in the later blocks of trials in a particular session, and c) more frequently by WKY 100% rats (see **Figure 3**).

**Figure 4:**
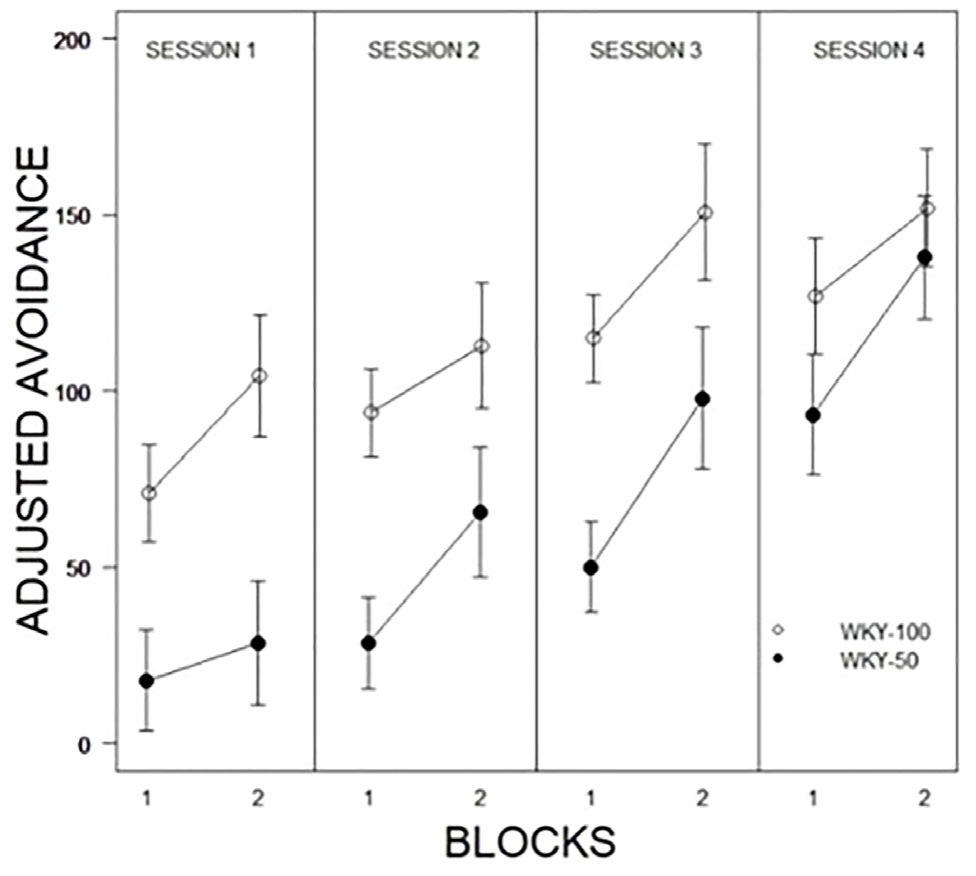
Comparison of adjusted avoidance performance as a function of partial predictability in WKY rats as a function of 10-trial blocks with training sessions. WKY-100 rats expressed avoidance to a higher degree than WKY-50 rats in each training session except the last. Legend is contained within the figure.

Of interest was accounting for the predictability of shock in acquisition and expression of avoidance. For the partial predictability groups, 50% of the trials would end with a shock if the rat did not emit an avoidance; thus, experience with the CS-US contingency depended in part on the behavior of the rat. Therefore, non-avoidance trials represent the actual experience of the rats with the CS-US contingency. Adjusted avoidance was calculated as a percent of experiences with the CS/US contingency during the first 10 trials of the first session of training. In addition to the main effects of Blocks, Sessions, and Predictability Schedule, a Predictability Schedule × Sessions, F(2.4, 73.8) = 3.1, p = .03, interaction was apparent (see **Figure 4**). Thus, by session 4, the advantage of the 100% group had ameliorated when the reduced number of pairings experienced early in training were taken into account.

## Discussion

A considerable amount of research has supported the proposal that behaviorally inhibited WKY rats are an important model for the development of anxiety disorders (19, 23, 26, 48, 50). In the current study, we extended work with WKY rats and avoidance to include a degree of uncertainty regarding the contingency between the CS and US with a schedule of partial predictability, in which one-half of the trials included a CS tone but no US shock. The major findings of this study fit with prior findings and provide further understanding of how WKY rats learn to make lever press avoidance responses in the face of uncertainty. First, WKY rats exhibited BI in an open field test. Second, WKY rats acquired avoidance more rapidly and at higher asymptotic levels than SD rats. Third, WKY rats were able to acquire robust levels of avoidance behavior with a 50% tone-only partial predictability schedule, whereas SD rats did not acquire avoidance with the partial predictability schedule. Fourth, adjusted avoidance (accounting for CS-US pairings experienced early in training), indicated that WKY rats in the partial predictability condition reached the same level of avoidance by the end of training as did WKY rats in the 100% CS-US condition. Fifth, WKY rats showed significantly more non-reinforced lever-press responding during the safety period inter-trial interval. These findings will be interpreted in the context of prior work and application to anxiety disorders and PTSD.

WKY rats expressed BI as confirmed by inhibited performance in the open field test when compared to SD rats. Specifically, WKY rats had longer latencies to leave the center and entered fewer sectors than did SD controls. This finding fits with studies from multiple labs that have demonstrated the behaviorally inhibited phenotype of the WKY rat (17, 19–21). Clearly, given the importance of BI as one key vulnerability factor for stress and anxiety disorders in humans [for review see (39, 40)], the WKY rat provides an excellent opportunity for behavioral and neural examination of factors that could contribute to the development of these disorders.

As expected from previous work, behaviorally inhibited WKY rats exhibited enhanced avoidance acquisition as compared to non-inhibited SD controls (19, 23, 25, 26, 48). Specifically, WKY rats acquired avoidance responding faster and to a greater degree than SD controls. The expression of avoidance behavior is a key factor in the development and diagnosis of PTSD (51). Acquisition of avoidance is a key vulnerability factor that appears to be expressed in behaviorally inhibited organisms (39, 40).

The major aim of the current study was to examine how WKY and SD rats would acquire avoidance with a schedule of partial predictability in which an element of uncertainty was introduced as to the probability of the aversive stimulus (i.e., the foot shock) following a tone. Additionally, if avoidance could be acquired on such a schedule, would the WKY rats continue to show superior avoidance as compared to SD rats. We assessed the effects of a 50% tone-alone schedule of partial predictability consisting of 10 tone-alone trials intermixed with 10 tone-shock trials during training. Both WKY and SD rats exhibited reduced avoidance responding in the 50% tone-alone condition as compared to the performance of each strain in the standard 100% tone-shock condition. This finding is consistent with the results of partial reinforcement studies of avoidance learning (41, 43, 52) as well as classical conditioning studies of schedules of partial reinforcement (32, 53–59). However, WKY rats in the 50% tone-alone condition outperformed SD rats in the 100% tone-shock condition, while SD rats in the 50% tone-alone condition did not acquire significant levels of avoidance responses and tended to escape rather than avoid the shock. WKY rats exhibited enhanced avoidance acquisition even when only one-half of the tone trials were paired with the foot shock reinforcement. This was similar to the findings of enhanced eyeblink conditioning with schedules of 50% tone-alone partial reinforcement in BI individuals (32) and individuals reporting PTSD symptoms (35). The robust avoidance learning of WKY rats on the 50% tone-alone schedule clearly shows the pervasiveness of avoidance in this strain. Some might describe the performance of SD rats in the 50% tone-alone condition as a failure to learn to avoid. However, based on the reduced contingency between the tone and shock, we suggest that this result can also be interpreted as the SD rats expressing resilience in the face of a shock that only followed a tone one-half of the time. An examination of the escape data for the SD rats on the 50% tone-alone schedule revealed that they were effectively making escapes (data not shown). Their strategy appears to have been to wait for the presence of the aversive stimulus and then respond, in contrast to the WKY rats which appear to be driven by the expectation of the aversive event. Thus, we suggest that the enhanced performance WKY rats in the 50% tone-alone schedule was indeed the expression of pathological responding to an uncertain situation, and the non-avoidant escaping behavior of the SD rats in the 50% tone-alone schedule was a resilient strategy in the face of uncertainty.

Nonreinforced lever-presses that occur during the safety period have been interpreted as perseverative responding that is a measure of internal drive for harm avoidance (19). Beck and colleagues demonstrated that nonreinforced responding during the safety period reveals both behaviorally inhibited and female sex vulnerabilities when using the contingent warning signal design (48). Our current results support these previous findings. Behaviorally inhibited WKY rats performed more non-reinforced lever-presses than non-inhibited SD rats. This was due primarily to a higher number of non-reinforced lever-presses by WKY rats in the 100% tone-shock condition. This would be expected, as Beck and colleagues demonstrated that nonreinforced responding was greater following escape performance (48). Our results suggest that, in the noncontingent design, WKY rats on a 100% tone-shock schedule show more nonreinforced safety period responding than SD rats. The 50% tone-alone schedule did not appear to produce as much nonreinforced responding, perhaps due to less opportunity for escape responding. The strategy of the WKY rats appears to be to make a bar press to the tone if there is any chance a shock will occur on that trial, whereas the SD rats appear to wait and see if the shock will occur if there is any chance the shock might not occur on that trial, which fits with the avoidant tendencies of the WKY rats.

The current work focused on the effects of a schedule of partial predictability that introduced an element of uncertainty into the signaled lever-press avoidance task. As described previously, intolerance of uncertainty plays a role in anxiety disorders and PTSD. In a review, Grupe and Nitchske described five mechanisms through which uncertainty can affect PTSD (60). These mechanisms included behavioral and cognitive avoidance, inflated estimates of threat cost and probability, increased threat attention and hypervigilance, heightened reactivity to threat conditions, and deficient safety learning. These mechanisms can explain some of our current findings as well as previous findings with WKY rats expressing enhanced avoidance and associative learning. Obviously, WKY rats exhibit enhanced behavioral avoidance in that they acquire avoidance responses faster and to a greater degree than SD rats in both the 100% tone-shock and 50% tone-alone conditions. The additional finding that WKY rats responded more to a 50% tone-alone training condition than SD rats can be explained by inflated estimates of threat cost and probability, increased threat attention, and hypervigilance, as well as heightened reactivity to threat conditions. Even though the shock only followed the tone one-half of the time in the partial predictability condition, the WKY rats avoided at higher rates, while SD rats did not acquire avoidance, opting rather for an escape strategy. More so, the WKY rats appear to inflate estimates of threat cost and probability, be hypervigilant, and over-react to threat conditions so much that their performance in the 50% tone-alone condition surpassed the performance of SD rats in the 100% tone-shock condition.

There is evidence of deficient safety learning in WKY rats in the lever press avoidance task in that, while male WKY rats were facilitated in avoidance learning by the addition of this safety signal, female WKY rats and both male and female SD rats did not exhibit facilitated avoidance with the additional safety signal (61). However, female WKY rats extinguished faster when the safety signal was removed. Beck and colleagues theorized that the flashing light during the ITI did not act as a safety signal, but rather as an occasion setter by producing a mild increase in arousal or attention to the forthcoming tone warning signal (61). In a subsequent study, male SD and WKY rats were tested on probe trials in which warning and safety signals were presented together after acquisition of an avoidance response (62). On these probe trials, SD rats decreased bar presses, which was interpreted as responding to the safety signal, while WKY rats increased bar presses, which was interpreted as responding to the warning signal.

Discrete trial avoidance is, in part, distinguished by the presence of a signal paired with shock; presence of the CS demarcates trials and provides a structured time interval for avoidance. Inasmuch as a bar press is not among species-specific defense reactions, responses are instrumentally acquired with a number of situational properties affecting expression (63). In most of the previous avoidance studies with WKY rats, a bar press avoidance resulted in the contingent termination of the CS and prevention of the shock. Avcu and colleagues used a noncontingent design, where the CS was delivered for 60 s regardless of whether an avoidance response was made (23). Further, the CS never overlapped with the shock delivery. Both the results of this prior study (23) and our current results demonstrated that rats can acquire avoidance when the termination of the CS is not contingent with the avoidance bar press. Both studies also demonstrated the superiority of the WKY rats in acquiring avoidance in the noncontingent CS design. The greater expression of avoidance is likely reflective of the threat properties of the CS and the reinforcement value in its cessation. Further, avoidance knowledge is acquired but not expressed with a short duration CS that is expressed more prominently with a longer CS (24).

Discrete trial avoidance is affected by the reinforcement contingencies. The two basic response contingencies in avoidance are defined by whether the requisite response is emitted or not and whether the aversive stimulus is delivered or not (64, 65). A partial reinforcement schedule delivers shock on some trials in which a response occurs, thus intermixing negative reinforcement and punishment. In a series of articles (41, 43, 52), Davenport, Olsen, and colleagues demonstrated that avoidance responses can be acquired with 50% partial reinforcement albeit at a somewhat slower rate. However, degrading the signal properties with regard to the presence or absence of shock has not, to our knowledge, been studied in discrete trial avoidance. Accordingly, the effects of partial predictability on avoidance acquisition of SD rats bears some comment. Under conditions of a noncontingent CS (as studied herein), the reinforcing properties of avoidance are apparent inasmuch as a few shock omissions will feedforward to sustained levels of avoidance expression. This pattern was evident in female SD rats with 100% pairing. An interesting side note of the behavior of SD rats is that with both predictability and control of shock established, female SD rats balance avoidance and escape responses, as opposed to avoiding the shocks altogether. This seeming preference for escape in the face of effective avoidance provides an operational definition of resiliency. To the degree such resiliency stands up in the face of more intense shock is an interesting question to explore. The partial predictability schedule severely degraded avoidance acquisition. Inspection of the records suggest that for SD rats, avoidance responses in the face of partial predictability were not reinforcing; there were few trials in which an avoidance was repeated from trial to trial once expressed. Under these conditions, escape is preferred. Partial predictability contrasts with partial reinforcement in that in the former, avoidance is not expressed, and in the latter, avoidance is expressed as asymptotic performance to a similar degree as full reinforcement.

Consistent with earlier studies (19, 23, 24), WKY rats expressed avoidance to a higher degree than SD rats. Avoidance expression was accompanied by increased nonreinforced responding, ITRs reduced over sessions in those trained with a full contingency. On face, the partial predictability protocol also degraded the avoidance expression of WKY rats. WKY rats trained with the partial predictability protocol exhibited fewer avoidance responses and nonreinforced responses compared to WKY rats trained with a full predictability schedule. With a straightforward analysis of avoidance, expression was substantially curtailed by the reduction in CS-US pairings. The reduced expression of avoidance in the face of partial predictability is seemingly counter to enhanced associativity in previous work. While expression under partial predictability was not to the degree of full predictability, accounting for the reduced numbers of CS-US pairings between those WKY rats trained with partial predictability and those with full predictability ameliorates the apparent differences in rates of expression by the last session of training. Given this adjustment, the otherwise slower expression in the first two sessions gives way to similar rates in the last session of training. This analysis suggests that expression of avoidance by WKY rats during partial predictability develops to a level at least on par with full predictability.

Nonetheless, the pattern of behavior of WKY rats in partial predictability is clearly distinguishable from SD rats which displayed a preference for escape under the same conditions. Reminiscent of findings in classical conditioning in humans expressing BI (32) and active duty military expressing PTSD symptoms (35), degraded predictability enhanced the power to detect strain differences in acquisition. The enhanced sensitivity is not readily apparent when comparing avoidance performance; the degree of difference between the strains in performance on full and partial predictability schedules was similar, hence the main effects of strain and partial predictability schedule. However, using avoidance performance during the fourth session to classify strain using receiver operator curves (ROCs) shows that with full predictability, strain classification based on avoidance performance is excellent (.89 ± .06), whereas classification with partial predictability is outstanding (.96 ± .04), which compares favorably to predictability in the open field activity (.98 ± .01).

A learning diathesis model of anxiety posits associative biases, representing a vulnerability to develop anxiety disorders *via* interaction with environmental or emotional stressors. As a core feature of anxiety disorders and PTSD, avoidance is acquired. Biases could be apparent as a greater sensitivity to acquire avoidance, to express avoidance under similar acquisition conditions, or resistance to extinction. Here, WKY rats display the former, a bias to acquire avoidance under conditions not normally supported. WKY rats acquired avoidance during partial predictability, conditions more suited to escape. Further, ROC analysis suggests that active coping with stressors with partial predictability is as strong a strain characteristic as inhibited temperament.

## Limitations and Future Work

The current study has the limitation that only female WKY and SD rats were tested. Based on the previous findings that females express higher levels of avoidance than males, and the expectation that the partial predictability manipulation would reduce avoidance acquisition, we elected to test females exclusively. However, based on prior sex differences in WKY rats, a follow-up study replicating the current work with both males and females would determine if the same effects for WKY and SD male rats are evident. There is some evidence that uncertainty has more of a negative impact on the action of females in a non-clinical sample as compared to males (66). However, several clinical studies of uncertainty did not find a sex effect (67–71). Future work exploring sex differences in uncertain conditions with WKY rats and behaviorally inhibited humans could contribute to our understanding of how sex, avoidance, and uncertainty interact in psychopathologies.

Another limitation of the current study is that it only involved acquisition of avoidance responses with the schedule of partial predictability and did not assess extinction with CS-alone trials. In addition to enhanced acquisition, WKY rats exhibit a resistance to extinction when the tone, but not the shock, is delivered (19, 25). The pervasiveness of avoidance, as observed in the current study, has been suggested as a key factor in why extinction is reduced in WKY rats [e.g., (19)]. Slowed extinction has also been reported for WKY rats in an eyeblink conditioning task (26). There is a long history in human eyeblink conditioning studies of CS-alone training producing a slowing of extinction when CS-alone trials are presented following CR acquisition to a schedule of partial reinforcement that included CS-alone trials. This slowed extinction is termed a partial reinforcement extinction effect or PREE (58, 72–74). More recently, Allen and colleagues found that behaviorally inhibited individuals exhibited slower extinction than non-inhibited individuals when training was switched from 50% CS-alone training to 100% CS-alone extinction training (32). This finding was further supported by slowed extinction for behaviorally inhibited military personnel self-reporting PTSD symptoms (35). The finding that extinction was slowed in behaviorally inhibited individuals is important in that slowing of extinction is commonly seen in individuals with anxiety disorders and exposure (i.e., extinction) training is commonly used for PTSD and anxiety disorders (75). A better understanding of why WKY rats and behaviorally inhibited humans as well as individuals with PTSD have trouble extinguishing may allow us to improve the training schedule in a course of prolonged exposure therapy (PE), thus improving outcomes. Equally important might be exploring why resilient individuals who experience a trauma but do not develop PTSD are able to extinguish more easily than those individuals who do develop PTSD. These insights into vulnerability and resilience derived from studies with WKY and SD rats could be applied to help patients with PTSD extinguish their own trauma-related associations.

Other schedules of partial reinforcement can also be tested with WKY and SD rats in the future. Davenport, Olsen, and colleagues (41, 43, 52) showed that avoidance responses are acquired at a slower rate with 50% partial reinforcement training in which CS-US trials were interspersed with trials in which responses during the CS were ineffective, resulting in US experience. Future work should examine how WKY and SD rats respond in this sort of partial reinforcement training in which responses to the CS fail to prevent the US. In a human BI study of partial reinforcement (32), Allen and colleagues reported that a schedule with 50% US air puff trials produced greater enhancement of eyeblink conditioning than the 50% CS-alone schedule used in the current study. In addition, the inclusion of US-alone trials did not interfere with acquisition of conditioned eyeblinks (32, 59). It would be of interest to test bar press avoidance in WKY and SD rats with a schedule in which un-signaled shocks are inter-mixed into tone-shock training. Studies with these two versions of partial reinforcement would provide further evidence for how behaviorally inhibited WKY rats and non-inhibited SD rats respond in uncertain learning situations that are suboptimal as compared to 100% CS-US paired training. In addition, these findings of partial predictability can be tested with humans expressing vulnerability to anxiety or anxious symptomology such as has been done with partial reinforcement (44).

Future work can also test partial predictability effects with WKY and SD rats in other avoidance tasks. Avoidance in the context of anxiety disorders and PTSD involves several forms, whether motivated by external events or experiences (behavioral avoidance) or internal concepts and affective states (experiential avoidance and emotional avoidance). In humans, avoidance can involve active forms of coping such as behavioral avoidance and procrastination or more inactive forms of coping such as repression and behavioral disengagement. In animal models, avoidance is characterized as being either passive (withholding a response to avoid an aversive stimulus) or active (performing an arbitrary response to prevent an aversive stimulus). One strength of the current experimental design with WKY rats is that they express both passive and active avoidance to an exaggerated degree. Avoidance by lack of action (related to BI behavior of WKYs in open field task) to avoid a novel situation and an active action (bar press) to avoid an aversive foot shock. Another avoidance task that involves bar pressing and both expression and inhibition of a response has been developed by Quirk and colleagues (76, 77). In this task, a rat is trained to lever press to receive a food reward. A warning signal (light) occurs that signals an impending foot shock which can be avoided by stepping onto a platform. The rat is performing an active avoidance response (stepping onto a platform) but is also ceasing its normal lever pressing for an appetitive reward. Examination of the effects of partial predictability with WKY and SD rats on this task would be interesting to determine how the strains would balance appetitive and avoidant responding.

### Conclusions

The major finding of the current study was that behaviorally inhibited WKY rats trained with a 50% CS-alone schedule of partial predictability outperformed non-inhibited SD rats trained with a 100% CS-US schedule. More specifically, WKY rats in the partial predictability condition did reach the same level of avoidance as rats in the 100% tone-shock condition when early experiences with CS-US paired trials were taken into account. This finding is interpreted as WKY rats being driven by the expectation of shock while non-inhibited rats are driven by the presence of shock. The findings of enhanced avoidance learning in behaviorally inhibited rats are consistent with enhanced eyeblink conditioning exhibited by behaviorally inhibited individuals trained with a schedule of partial reinforcement. Taken together, these studies with some degree of uncertainty such as partial reinforcement or partial predictability may be more ecologically valid than training with exclusively CS-US paired trials in that rarely is an aversive event in the real world predicted by some signal or cue 100% of the time. The present findings support the future use of these partial schedules to investigate the role of uncertainty in various forms of learning. A learning diathesis model of anxiety posits associative biases, representing a vulnerability to develop anxiety disorders *via* interaction with environmental or emotional stressors. As a core feature of anxiety disorders and PTSD, avoidance is acquired. Biases could be apparent as a greater sensitivity to acquire avoidance or express avoidance under non-optimal conditions or as a resistance to extinction. Here, WKY rats display the former, a bias to acquire avoidance under conditions not normally supported. WKY rats acquired avoidance during partial predictability, conditions more suited to escape. Further, ROC analysis suggests that active coping with stressors with partial predictability is as strong a strain characteristic as inhibited temperament.

Additionally, our findings support the utility of WKY rats as a model of BI as well as the use of schedules of partial reinforcement which can be used to explore the effects of uncertainty in PTSD. Animal models such as the WKY rat provide the opportunity to test parametric manipulations of learning conditions including acquisition as well as extinction and allow for more invasive manipulations of neural substrates that may underlie psychopathologies such as PTSD and anxiety disorders. Continued parallel studies in humans and rodents expressing BI are needed to further explore how personality temperaments may increase vulnerabilities for the development of PTSD and anxiety disorders through altered classical and instrumental conditioning, especially in conditions that include some element of uncertainty.

## Data Availability Statement

The raw data supporting the conclusions of this article will be made available by the authors, without undue reservation.

## Ethics Statement

The animal study was reviewed and approved by Institutional Animal Care and Use Committee, Carthage College.

## Author Contributions

DM, MA, and RS contributed to the design of the study. DM collected the data. DM and RS contributed to the data analysis. DM, MA, and RS contributed to the preparation and editing of the manuscript.

## Funding

Research supported by the Stress and Motivated Behavior Institute, Syracuse, NY, and Carthage College, Kenosha, WI.

## Conflict of Interest

The authors declare that the research was conducted in the absence of any commercial or financial relationships that could be construed as a potential conflict of interest.
